# Adenovirus vector-based multi-epitope vaccine provides partial protection against H5, H7, and H9 avian influenza viruses

**DOI:** 10.1371/journal.pone.0186244

**Published:** 2017-10-12

**Authors:** Ahmed O. Hassan, Omar Amen, Ekramy E. Sayedahmed, Sai V. Vemula, Samuel Amoah, Ian York, Shivaprakash Gangappa, Suryaprakash Sambhara, Suresh K. Mittal

**Affiliations:** 1 Department of Comparative Pathobiology and Purdue Institute for Inflammation, Immunology, and Infectious Disease, Purdue University, West Lafayette, IN, United States of America; 2 Poultry Diseases Department, Faculty of Veterinary Medicine, Assiut University, Assiut, Egypt; 3 Influenza Division, Centers for Disease Control and Prevention, Atlanta, GA, United States of America; Center for Inflammation, Immunity & Infection, Institute for Biomedical Sciences, UNITED STATES

## Abstract

The emergence of H5, H7, and H9 avian influenza virus subtypes in humans reveals their pandemic potential. Although human-to-human transmission has been limited, the genetic reassortment of the avian and human/porcine influenza viruses or mutations in some of the genes resulting in virus replication in the upper respiratory tract of humans could generate novel pandemic influenza viruses. Current vaccines do not provide cross protection against antigenically distinct strains of the H5, H7, and H9 influenza viruses. Therefore, newer vaccine approaches are needed to overcome these potential threats. We developed an egg-independent, adenovirus vector-based, multi-epitope (ME) vaccine approach using the relatively conserved immunogenic domains of the H5N1 influenza virus [M2 ectodomain (M2e), hemagglutinin (HA) fusion domain (HFD), T-cell epitope of nucleoprotein (TNP). and HA α-helix domain (HαD)]. Our ME vaccine induced humoral and cell-mediated immune responses and caused a significant reduction in the viral loads in the lungs of vaccinated mice that were challenged with antigenically distinct H5, H7, or H9 avian influenza viruses. These results suggest that our ME vaccine approach provided broad protection against the avian influenza viruses. Further improvement of this vaccine will lead to a pre-pandemic vaccine that may lower morbidity, hinder transmission, and prevent mortality in a pandemic situation before a strain-matched vaccine becomes available.

## Introduction

Human infections caused by H5, H7, and H9 avian influenza virus subtypes provide evidence of these strains’ pandemic potential [1, 2]. The highly pathogenic avian influenza (HPAI) H5N1 virus initially emerged in China, and subsequently spread to over 60 countries on three continents. H5N1 is currently endemic among the poultry populations in South East Asia and Africa. Over 859 confirmed human infections of H5N1 and 453 deaths (a case fatality rate of approximately 52%) have been reported since 2003 [3].

In 2013, a new H7N9 avian influenza virus strain emerged in China [4] and has since caused more than 1589 human infections and 616 deaths [5]. A H7N9 virus isolate (A/Shanghai/2/2013) was found to efficiently infect ferrets and pigs with virus shedding, and the virus was efficiently transmitted from infected ferrets to uninfected ferrets via direct contact [6, 7]. Qi et al. suggest that the H7N9 virus has the potential to spread among human populations [8]. However, structural analysis indicates that H7N9 hemagglutinin (HA) characteristics are similar to avian HA, including a strong binding affinity to α2-3-linked glycans [9]. These similarities offer evidence that current H7N9 viruses are poorly adapted for human-to-human dissemination. Still, there exists a possibility for H7N9 viruses to acquire the capability to be the next pandemic virus. Human cases of other H7 influenza viruses (e.g., H7N2, H7N3, and H7N7) linked to poultry outbreaks have been reported in the Netherlands, Canada, Mexico, Italy, the U.S., and the U.K. [10, 11]. Moreover, H9N2 viruses have caused human infections in Hong Kong, Bangladesh, and South Korea [10, 12, 13]. Although H5N1, H7N7, H7N9, and H9N2 avian influenza viruses are not readily transmissible among humans, it is possible that any of these viruses could reassort with a circulating human influenza strain, or acquire the required mutations of the HA gene segment, and able to attach to human-like α2-6-sialic acid receptors [14, 15]. This possibility is very likely to result in a novel pandemic influenza strain.

For pandemic preparedness purposes, H5N1, H7N7, and H9N2 virus vaccines have been developed and clinically evaluated. However, the inactivated or subunit H5 vaccines were found to be low immunogenic, which required the administration of an adjuvant, higher HA doses, or the administration of multiple doses in preclinical and clinical trials [16–18]. Furthermore, the diversity of the H5N1 virus has expanded exponentially in the past few years. The virus now has evolved into genetically distinct clades and subclades, which makes the development of a strain-matched vaccine using traditional methods extremely challenging. The development efforts for H7 and H9 influenza virus vaccines pose similar concerns [19, 20]. Recently, a vaccine against H7N1 that was developed in cell culture was proven to be poorly immunogenic in humans, except when administered with an adjuvant [20]. In addition, a study has shown that the H9N2 vaccine failed to provide cross-reactivity against heterologous viruses from a different clade in human clinical trials, despite the use of an MF59 adjuvant in conjugation with the vaccine [21]. Hence, there is a pressing need for the development of a pre-pandemic influenza vaccine that can induce both humoral and cellular immune responses and provide broad protection against a wide range of avian influenza viruses currently emerging from avian reservoirs.

Adenovirus (Ad) vector-based vaccines induce excellent humoral and cell-mediated immune (CMI) responses [22, 23] due to the adjuvant-like ability of Ad vectors to stimulate the innate immune system through both Toll-like receptor (TLR)-dependent and TLR-independent pathways [24, 25]. Ad vector-based influenza vaccines have shown excellent potential in both animal models [26–28] and clinical trials [29, 30]. Our immunogenicity and protective efficacy studies in mice demonstrate that Ad vector-based vaccines provide complete protection against homologous and antigenically distinct influenza virus strains [28].

The M2 ectodomain (M2e) [31–35], HA fusion domain (HFD) [36–39], T-cell epitope of nucleoprotein [NP] (TNP) [40–42], and HA α-helix domain (HαD) [36–39, 43–47] have been identified as relatively conserved immunogenic domains with the potential to provide cross-protection against influenza viruses. These epitopes are either hidden within the native protein structure or poorly immunogenic without the use of a strong adjuvant. We hypothesize that using these immunogenic domains and their expression in an Ad vector will result in the induction of optimal immune responses against cross-protective epitopes. The present study describes the Ad vector-based, multi-epitope (ME) vaccine approach for broadening protective immunity against the H5, H7, and H9 avian influenza virus subtypes. An Ad vector expressing ME (M2e, HFD, TNP and HαD) of a H5N1 avian influenza virus was generated and evaluated for its immunogenicity and protective efficacy in a mouse model. Immunogenicity studies demonstrated that M2e, HFD, TNP, and HαD epitopes were recognized in the Ad vector formulation. In order to mimic a pandemic situation, antigenically distinct H5N2, H7N9, and H9N2 avian influenza viruses were used to evaluate our vaccine’s protection efficacy. The immunogenicity and protection study results suggest that the development of a pre-pandemic influenza vaccine using the relatively conserved immunogenic epitope approach is possible.

## Materials and methods

### Cells and viruses

293 (human embryonic kidney cells that express E1 gene products of human Ad-C5), 293Cre (293 cell line that expresses Cre recombinase), MDCK (Madin-Darby canine kidney), and BHH2C (bovine-human hybrid clone 2C) [48] cell lines were used for this study. These cells were grown as monolayer cultures in minimum essential medium (MEM) (Life Technologies, Gaithersburg, MD) supplemented with 10% reconstituted fetal bovine serum (Hyclone, Logan, UT) and gentamycin (50 μg/ml).

The following low pathogenic influenza A viruses were used in this study: A/chukar/MN/14951-7/1998(H5N2), A/goose/Nebraska/17097/2011(H7N9), and A/Hong Kong/1073/1999(H9N2). These influenza viruses were grown in embryonated hen eggs and were titrated in eggs, MDCK, or both. These viruses will not cause significant morbidity or mortality in mice since they were not mouse-adapted. Therefore, the lung virus titers following challenge will be the best measurable parameter to evaluate the vaccine’s efficacy.

### Generation of human Ad replication defective vectors

Human Ad vectors were generated in 293Cre cells using Cre-recombinase site-specific recombination [49]. The construction of Ad-H5HA, Ad-H7HA, and Ad-H9HA, each containing a full-length coding region of H5 HA of A/Vietnam/1203/2004(H5N1), H7 HA of A/Netherlands/219/2003(H7N7), or H9 HA of A/chicken/Hong Kong/G9/1997(H9N2), respectively as previously described [50, 51]. The polybasic cleavage sites of full HA genes of H5 and H7 were modified to match the low pathogenic strains. The generation of empty vector Ad-ΔE1E3 (E1 & E3 deleted Ad-C5) is described elsewhere [52].

For the ME vaccine, the following M2e, HFD, TNP, and HαD domains of H5N1 (A/Vietnam/1203/2004) were separated by the AlaAlaAla, GlyGlyGly and GlyProGlyProGly linkers, respectively:

M2e: MSLLTEVETPTRNEWECRCSDSSD;
HFD: GLFGAIAGFIEGGW;
TNP: TYQRTRALVR;
HαD: RIENLNKKMEDGFLDVWTYNAELLVLMENERTLDFHDSNVKNLYDKVRLQLRD
NA.

**Fig 1:**
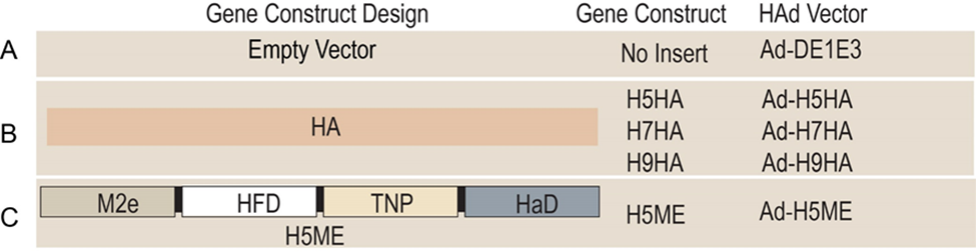
Schematic diagram of adenovirus (Ad) vector constructs used in the study. (A) Ad-ΔE1E3; human adenovirus type C5 (Ad-C5) empty vector. (B) Ad-H5HA, Ad-H7HA and Ad-H9HA; Ad-C5 vector containing a full-length coding region of H5 hemagglutinin (HA) of A/Vietnam/1203/04(H5N1), H7 HA of A/Netherlands/219/2003(H7N7) or H9 HA of A/chicken/Hong Kong/G9/1997(H9N2), respectively. (C) Ad-H5ME, Ad-C5 vector containing multi-epitope (ME) gene construct of A/Vietnam/1203/ 2004(H5N1) that contains the extracellular domain of M2 (M2e), fusion domain of HA (HFD), T-cell epitope of nucleoprotein [NP] (TNP), and alpha helix domain of HA (HαD).

The H5N1 ME (H5ME) gene cassette was synthesized commercially. The construct structures and their names are shown in Fig 1. The Ad vector containing H5ME was generated in 293Cre cells using Cre-recombinase site-specific recombination. The gene construct was under the control of the human cytomegalovirus (HCMV) immediate early promotor and the bovine growth hormone (BGH) polyadenylation signal. All Ad constructs were purified using cesium chloride density-gradient ultracentrifugation [53] and titrated in BHH2C cells [48].

### Animal inoculation and protection studies

All animal experiments were conducted in a USDA-approved BSL-2+ facility at Purdue University with the approval and in accordance with the guidelines of the Institutional Biosafety Committee and Institutional Animal Care and Use Committee. Six-to-eight-week-old female BALB/c mice (purchased from Harlan Sprague Dawley Inc., Indianapolis) were used to evaluate the breadth of immune responses and protection efficacy. Immunization and challenge experiments were conducted in two phases. During the first phase, five groups of mice (7 animals/group) were immunized via intramuscular inoculation of 10^8^ PFU of each of Ad vectors as follows: Ad-H5HA, Ad-H7HA, Ad-H9HA, Ad-H5ME, or empty vector Ad-ΔE1E3. The immunization was administered twice within a three-week interval. The animals were anesthetized with ketamine-xylazine (90 mg/kg ketamine and 10 mg/kg xylazine) via intraperitoneal injections three weeks after the second immunization. Blood samples were collected via retro-orbital puncture to evaluate the humoral immune responses, and the spleens were collected upon euthanasia to evaluate the cell-mediated immune responses. The mice under anesthesia were euthanized using cervical dislocation and the thorax was perforated to ensure death. These procedures were in accordance with the recommendations of the American Veterinary Medical Association’s Panel on Euthanasia.

During the second phase, similar vaccinations as described for the first phase were followed with five animals in each group. Three weeks after the final vaccination, the animals were challenged intra-nasally with a 100 mouse infectious dose 50 (MID_50_) of A/chukar/MN/14951-7/1998(H5N2), A/goose/Nebraska/17097/2011(H7N9) or A/Hong Kong/1073/1999(H9N2). Three days after the challenge, the animals were euthanized under anesthesia with ketamine-xylazine (90 mg/kg ketamine and 10 mg/kg xylazine) via intraperitoneal injections, and the lungs were collected and then homogenized in 1 ml of sterile phosphate-buffered saline (PBS). A ten-fold serial dilution of the lung homogenates was used to infect either MDCK cells in 96-well plates (for H5N2 and H9N2 virus-challenged groups) or specific-pathogen free (SPF) embryonated hen eggs (for H7N9 virus-challenged groups). Following 72 h incubation at 37°C, the infected-cell supernatants or allantoic fluid samples were tested by hemagglutination assay (HA) using 0.5% turkey red blood cells (TRBC) to determine a 50% tissue culture infectious dose (TCID_50_) [for H5N2 and H9N2 virus-challenged groups] or 50% egg infectious dose (EID_50_) [for H7N9 virus-challenged groups] using the Reed-Muench method [54].

### Hemagglutination inhibition assay

The mouse serum samples were treated with receptor-destroying enzyme (RDE) [Denka Seiken, Tokyo, Japan] and incubated at 37°C for 20 h. Thereafter the samples were inactivated by incubation at 56°C for 30 min. Two-fold dilutions of RDE-treated sera were conducted in 96-well plates first with a 1:10 dilution and followed by four hemagglutination units (HAU) of each virus diluted in PBS. Following incubation at room temperature for 1 h, 0.5% TRBC was added and the plates were incubated at room temperature for 20 min to allow hemagglutination to occur. The hemagglutination inhibition (HI) antibody titer was determined as the reciprocal of the highest dilution that completely prevented TRBC from agglutination, and the data are presented as the geometric mean HI titers.

### Virus neutralization assays

The assays were performed as previously described [28]. Two-fold serial dilutions of heat-inactivated serum samples were mixed with 100 TCID_50_ or 100 EID_50_ of an influenza virus, and the mixture was incubated for 1 h at 37°C. The mixture was then added to MDCK cells in 96-well plates or injected into the allantoic fluid of embryonated hen eggs and incubated for 72 h at 37°C. Thereafter, the HA activities of the cell supernatants or allantoic fluid samples were detected by HA assay with 0.5% TRBC. The virus neutralization (VN) titer was determined as the reciprocal of the highest dilution that completely neutralized the virus, and the data are presented as the geometric mean VN titers.

### Enzyme-linked immunosorbent assay (ELISA)

ELISA was performed as described earlier [50, 55]. The M2e, HαD, and HFD peptides biotinylated at the N-terminus were commercially synthesized (Gen Script, Piscataway, NJ). 96-well ELISA plates (eBioscience, San Diego, CA) were coated with streptavidin and incubated overnight at 37°C. Then the biotinylated M2e, HαD, or HFD peptide (5 μg/ml) were added and incubated at room temperature for 1 h. After blocking the non-specific protein-binding sites with PBS containing 1% bovine serum albumin (BSA), diluted serum samples (1:50 dilution for M2e or HFD and 1:200 dilution for HαD) were added and incubated at room temperature for 3 h. During the standardization process, various dilutions of the serum samples for each peptide were used to determine the best serum dilution for each peptide. The horseradish peroxidase-conjugated goat anti-mouse IgG (Sigma-Aldrich, Inc., St. Louis, MO) at a dilution of 1:2000 was added to the wells, followed by 1 h incubation at room temperature. The plates were developed with a TMB substrate (Biolegend, San Diego, CA). The reaction was stopped by adding 1 M sulfuric acid, and the optical density measurements were taken at 450 nm using a microplate reader (Molecular Devices, Sunnyvale, CA).

### ELISpot assay

The assay was performed as previously described [28]. The spleens collected from mice following three weeks post-booster immunization were homogenized using cell strainers (Fisher Scientific, Hampton, NH). The red blood cells were lysed with the ACK lysis buffer (Lonza Walkersville, Inc., MD). The splenocytes were then resuspended in RPMI-1640 medium containing L-glutamine and 25mM HEPES (Gibco, Grand Island, NY), 10% reconstituted fetal bovine serum, nonessential amino acids, sodium pyruvate, β-mercaptoethanol, penicillin/streptomycin, and gentamycin. The viable cell count was determined by mixing splenocytes with 0.4% Trypan Blue stain (Fisher Scientific, Hampton, NH) and then counting the unstained live cells on a hemocytometer under a light microscope.

PVDF-backed 96-well microplates (Millipore, Billerica, MA) were coated overnight at 4°C with a monoclonal antibody for mouse interferon-gamma (INFγ) [BD Bioscience, San Jose, CA]. 10^6^ splenocytes (in RPMI-1640 medium with supplements) were incubated with either HA518 (IYSTVASSL) or NP147 (TYQRTRALV) peptides (H-2K^d^-restricted cytotoxic T lymphocyte (CTL) epitopes for HA and NP, respectively) for 60 h at 37°C in a CO_2_ incubator. As a positive control, the splenocytes were cultured in the presence of phorbol myristate acetate/ionomycin (Sigma-Aldrich, St. Louis, MO). The cells were removed by washing, and a biotinylated rat anti-mouse antibody specific for INFγ (BD Bioscience, San Jose, CA) was added. The plates were incubated overnight at 4°C. Following the addition of streptavidin-AP (Sigma-Aldrich, St. Louis, MO) and washing, a chromogen mixture of BCIP/NBT (Sigma-Aldrich, St. Louis, MO) was added for color development. The reaction was stopped by rinsing the plates with deionized water. The ELISpot plates were read using a Bioreader 5000 (BIOSYS, Miami, FL) to count the number of spot-forming units (SFU).

### Statistical analyses

For statistical significance determination, one- and two-way ANOVA tests were conducted with Dunnett's post-hoc analysis at a significance level of *p*<0.05.

## Results

### Generation of Ad vector expressing ME of H5N1 influenza virus

A Cre-recombinase site-specific recombination system was used to insert a coding sequence of the ME containing M2e, HFD, TNP, and HαD of H5N1 (H5ME) into the E1 region of Ad-C5 to generate Ad-H5ME (Fig 1). The recombinant virus showed cytopathic effects 7–10 days post-transfection of 293Cre cells. The construction and characterization of Ad-H5HA, Ad-H7HA, and Ad-H9HA have been previously described [50, 51].

### Induction of humoral immune responses against H5ME

**Fig 2:**
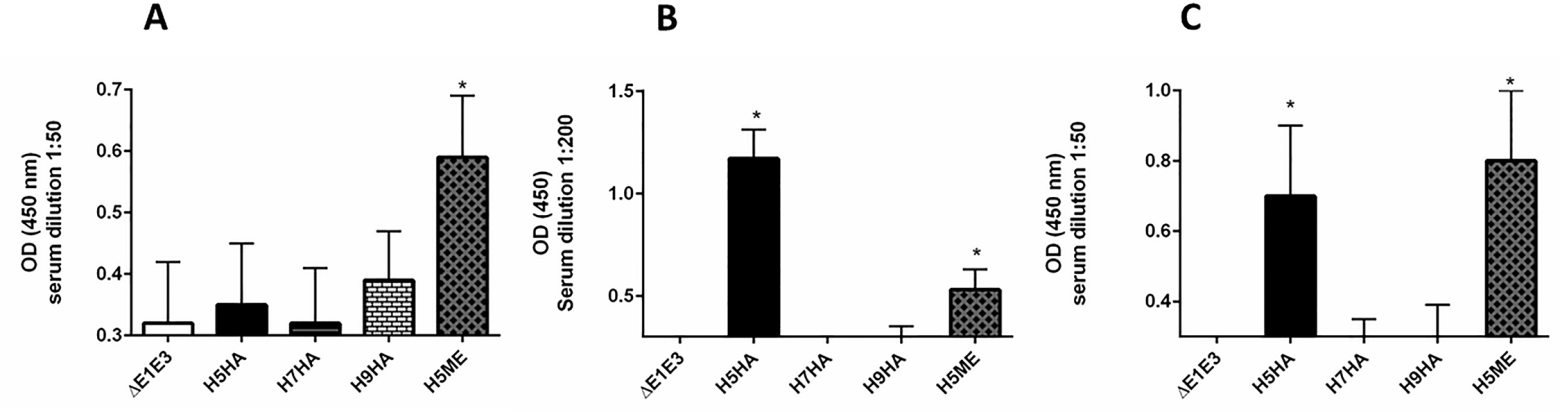
Influenza antigen-specific antibody responses generated after immunization with an Ad vector-based multi-epitope (ME) vaccine. Three weeks after the second inoculation, serum samples were collected from all animal groups as described in the material and methods section, and tested by ELISA for IgG antibody responses specific to M2e (A), HαD (B) or HFD (C). Data are represented as the mean ± standard deviation (SD) of the optical density (OD) readings. Based on OD values, M2e-, HαD- and HFD-specific IgG responses induced by H5ME were statistically significant (*P≤ 0.05 as compared to the ΔE1E3 control). H5HA, Ad-H5HA; H7HA, Ad-H7HA; H9HA, Ad-H9HA, H5ME, Ad-H5ME; ΔE1E3, Ad-ΔE1E3.

The mice were inoculated i.m. with various Ad vectors, as described in the Material and Methods section. Three weeks following the booster inoculation, serum samples were collected and assayed for M2e-, HαD-, or HFD-specific IgG by ELISA (Fig 2). In this study, we did not monitor the immunogenicity of the prime dose (single inoculation prior to the second dose of the vaccine); however, it seems that the immunization of mice with a single dose of Ad vector-based vaccine elicited protective immune responses, and the second inoculation resulted in measurable increases in immunogen-specific immune responses [56].

Mice vaccinated with Ad vector expressing H5ME (Ad-H5ME) showed high levels of M2e-specific IgG (Fig 2A). For HαD-specific IgG responses, mice vaccinated with Ad-H5ME as well as Ad-H5HA (vector containing the full H5HA gene) showed elevated levels of HαD-specific IgG (Fig 2B). Similarly, HFD-specific IgG responses increased in mice vaccinated with Ad-H5ME (Fig 2C).

The serum samples from immunized mice were also analyzed for HI titers to determine the presence of HA-specific antibodies against heterologous H5 (H5N2), H7 (H7N9), and H9 (H9N2) avian influenza viruses, as we have previously elucidated that Ad vectors elicit enhanced HI titers against homologous avian influenza viruses [50]. HI titers (320) against the H5N2 virus were observed in the Ad-H5HA-immunized group only (Table 1). Detectable HI activities (176) against the H7N9 virus were noticed in the Ad-H7HA-inoculated group only (Table 1). Similarly, HI antibody titers (44) against the H9N2 virus were detected in the Ad-H9HA-inoculated group only (Table 1).

**Table 1:** Hemagglutination inhibition (HI) and virus neutralization (VN) antibody titers in mice vaccinated with an Ad vector-based multi-epitope (ME) vaccine.

Vaccine group	H5N2		H7N9		H9N2	
	HI	VN	HI	VN	HI	VN
**ΔE1E3**	<10	<10	<10	<10	<10	<10
**H5HA**	320	246	<10	<10	<10	<10
**H7HA**	<10	<10	176	780	<10	<10
**H9HA**	<10	<10	<10	<10	44	31
**H5ME**	<10	<10	<10	<10	<10	<10

Mice (7 mice/group) were immunized twice with H5HA, H7HA, H9HA, H5ME or ΔE1E3 at an interval of three weeks. Three weeks after the final immunization, serum samples were collected for detecting HI and VN antibody titers. The titers are shown as the geometric mean titers. H5HA, Ad-H5HA; H7HA, Ad-H7HA; H9HA, Ad-H9HA,; H5ME, Ad-H5ME; ΔE1E3, Ad-ΔE1E3; H5N2, A/chukar/MN/145917/1998(H5N2); H7N9, A/goose/Nebraska/17097/2011(H7N9); H9N2, A/Hong Kong/1073/ 1999(H9N2).

The serum samples from various inoculated groups were also analyzed for neutralizing antibodies against the H5N2, H7N9, and H9N2 influenza viruses. Since the influenza viruses used for microneutralization assays have antigenically distinct HAs, lower levels of virus neutralizing antibody titers were anticipated depending on the differences between the H5, H7, and H9 HA sequences in the Ad vectors, and the HA sequences of the H5N2, H7N9, and H9N2 influenza viruses used for microneutralization assays. Mice vaccinated with Ad vectors containing the HA construct, Ad-H5HA, Ad-H7HA, or Ad-H9HA developed 246, 780, and 31 geometric mean VN titers against H5N2, H7N9, and H9N2 influenza viruses, respectively (Table 1). Noticeably, the Ad-H5ME-inoculated group did not develop HI or VN antibodies against H5N2, H7N9, or H9N2 viruses (Table 1).

### Induction of cell-mediated immune responses against H5ME

**Fig 3:**
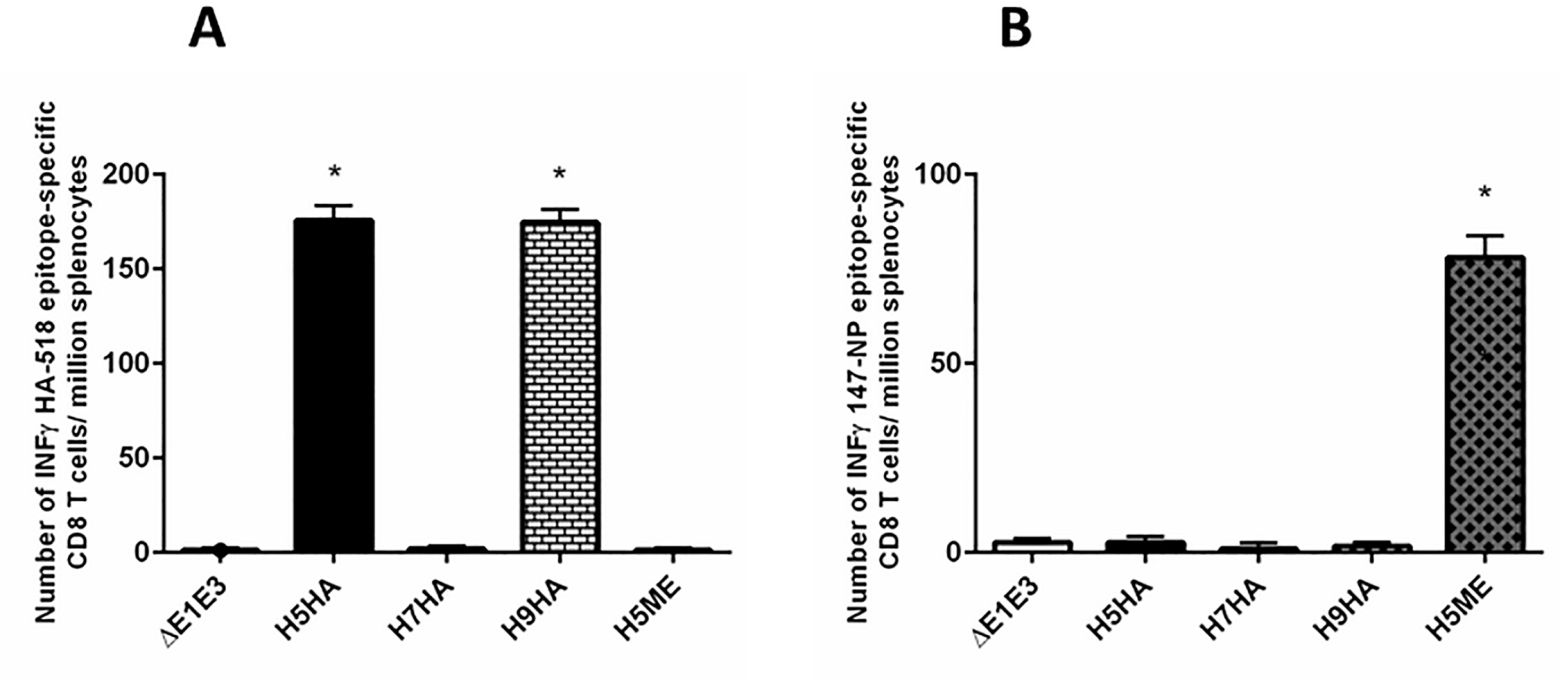
HA518 and NP147 epitope-specific IFNγ secreting CD8+ T cells in the spleens of vaccinated mice. Three weeks after the second inoculation, the spleens were collected from all animal groups after euthanizing the animals as described in the material and methods section. The splenocytes were evaluated for HA-specific (A) or NP-Specific (B) cell-mediated immune responses using INFγ-ELISpot assay. The data represent mean ± standard deviation (SD). The number of spot-forming units (SFU) induced by H5ME in NP-specific INFγ-ELISpot, and the number of SFU induced by H5HA or H9HA in HA-specific INFγ-ELISpot were found to be statistically significant (*P≤ 0.05 as compared to the ΔE1E3 control). H5HA, Ad-H5HA; H7HA, Ad-H7HA; H9HA, Ad-H9HA; H5ME, Ad-H5ME; ΔE1E3, Ad-ΔE1E3.

It is well known that CMI responses against influenza viruses play a role in virus clearance [57]. To evaluate the CMI responses against influenza in immunized mice, the spleens from Ad-vector inoculated groups were collected three weeks after the second immunization. The splenocytes were isolated and analyzed for HA- or NP-specific CMI responses following *in vitro* stimulation with HA518 or NP147 peptide, respectively, using an IFNγ-specific ELISpot assay. Due to the fact that HA518 epitope, a CD8 T-cell epitope, is conserved in H5HA and H9HA, but not in H7HA, the animal groups that received Ad-H5HA or Ad-H9HA showed higher numbers of INFγ-secreting HA518-specific CD8 T cells compared to the empty vector (Ad-ΔE1E3) control group (Fig 3A). These results are consistent with our previous findings [50]. Mice that were vaccinated with Ad-H5ME showed increased numbers of NP147-specific IFNγ-secreting CD8 T cells compared to the empty vector (Ad-ΔE1E3) control group (Fig 3B).

### Breadth of protection conferred by Ad vector-based ME vaccine following challenge with antigenically distinct H5N2, H7N9, or H9N2 influenza viruses

One of the important vaccine efficacy measures is the lung viral titers in vaccinated animals challenged with an influenza virus [28]. A significant decrease in lung viral titers is indicative of the vaccine protective efficacy and the reduction in the virus transmission. Ad vector-based vaccine efficacy in mice was evaluated by measuring the lung viral titers three days after challenges with a 100 MID_50_ of H5N2, H7N9, or H9N2 influenza virus. As expected, immunization with Ad-H5HA, Ad-H7HA, or Ad-H9HA resulted in complete protection with a homosubtypic virus only **(**Table 2**)**. Animal groups that were vaccinated with Ad-H5ME showed 1.5, 1.2, and 1.8 log reductions in the lung viral titers of antigenically distinct H5N2, H7N9, or H9N2 influenza virus, respectively, compared to the Ad-ΔE1E3-inoculated groups (Table 2).

**Table 2:** Lung viral titers in vaccinated mice after challenge with H5N2, H7N9 or H9N2 influenza viruses.

Vaccine group	Lung virus titer after H5N2 challenge, Log_10_ TCID_50_/ml	Lung virus titer after H7N9 challenge, Log_10_ EID_50_/ml	Lung virus titer after H9N2 challenge, Log_10_ TCID_50_/ml
**ΔE1E3**	3.9±0.57	4.9±0.27	6.2±0.5
**H5HA**	< 1.2*	4.5±0.27	5.8±0.62
**H7HA**	3.5+0.35	<1.2*	5.9±0.35
**H9HA**	3.7±0.43	4.7±0.54	< 1.2*
**H5ME**	2.4±0.32*	3.7±0.54*	4.4±0.6*

Mice (5 mice/group) were immunized twice with H5HA, H7HA, H9HA, H5ME, or ΔE1E3 at an interval of three weeks. Three weeks after the final immunization, mice were challenged with one of the following viruses: H5N2, H7N9 or H9N2. Three days after the challenged, mice were euthanized and the lungs were harvested to determine lung virus titers. The data are shown as mean Log_10_ TCID_50_±SD (for H9N2 and H5N2) or mean Log_10_ EID_50_±SD (for H7N9). The detection limit of lung viral titer was <1.2 Log_10_ TCID_50_/ml (for H9N2 and H5N2) or <1.2 Log_10_ EID_50_/ml (for H7N9) [indicated as <1.2].

*P≤ 0.05 as compared to the ΔE1E3 control. H5HA, Ad-H5HA; H7HA, Ad-H7HA; H9HA, Ad-H9HA,; H5ME, Ad-H5ME; ΔE1E3, Ad-ΔE1E3; TCID_50_, tissue culture infectious dose 50; EID_50_, egg infectious dose 50; SD, standard deviation; H5N2, A/chukar/MN/145917/1998(H5N2); H7N9, A/goose/Nebraska/17097/2011(H7N9); H9N2, A/Hong Kong/1073/ 1999(H9N2).

## Discussion

Incidences of human infections with H5N1, H7N7, H7N1, H7N3, H7N9, and H9N2 avian influenza virus subtypes have demonstrated their pandemic potential; however, the characteristics of a new pandemic influenza virus will be unknown until the time of the pandemic. The development of a pre-pandemic influenza vaccine seems to be one of the most effective measures of pandemic preparedness. The pre-pandemic influenza vaccine needs to provide broad cross-protection, given that it is almost impossible to predict if and when H5N1, H7N9, H9N2, or another influenza virus will acquire the characteristics of a pandemic virus. In this manuscript, our efforts were directed towards the generation of a broadly protective vaccine against H5, H7, and H9 influenza subtypes, with the anticipation that such a vaccine would significantly lower morbidity, hinder transmission, and prevent mortality in a pandemic situation before a strain-matched vaccine can be produced. Our vaccine could also prime human populations for improved immune responses in the aftermath of pandemic virus infections.

The relatively conserved influenza A virus epitopes have been investigated for their potential as immunogens to induce cross-protective immune responses. Vaccine strategies targeting relatively conserved domains, for example M2e and HA2, have the ability to induce cross-reactive humoral immune responses [36, 58]. Internal proteins containing relatively conserved T-cell epitopes (e.g., M1, PB1, and NP) were also investigated for their ability to induce cross-protective T-cell responses [59, 60]. In the present study, we attempted to combine the heterosubtypic CMI response against TNP and the cross-reactive (not necessarily cross-neutralizing) humoral immune responses against M2e, HFD, and HαD domains to provide broad protection against a potential H5N1, H7N9, or H9N2 avian influenza virus pandemic. The Ad vector system was used to deliver ME containing the relatively conserved B- and T-cell epitopes (M2e, HFD, TNP, and HαD) of H5N1 in a single vector formulation. To mimic an influenza pandemic, we purposely used three antigenically distinct wild type influenza viruses, A/chukar/MN/14951-7/1998(H5N2), A/goose/Nebraska/17097/2011(H7N9) and A/Hong Kong/1073/1999(H9N2), to represent the H5N1, H7N7, and H9N2 viruses, respectively.

In the present study, the Ad-H5ME expressing ME of H5N1 (H5ME) was recognized by the host immune system as evident with significant levels of M2e-, HFD-, and HαD-specific antibody responses as well as the T-cell response to the TNP epitope. As expected, Ad-H5ME did not induce H5N2-specific HI or VN antibodies in immunized animals. However, following challenges with H5N2, H7N9, or H9N2, there was approximately 1.5, 1.2, and 1.8 log reductions in lung virus titers, respectively, which is indicative of cross-protection. The levels of cross-reactive humoral immune responses could vary significantly depending on the HA subtype antigenic group (H5 and H9 in Group 1 and H7 in Group 2) [61]. The lower levels of cross protection against H7N9 compared to H5N2 or H9N2 in this study may be due to the antigenic differences between Groups 1 and 2.

The N terminal fusion domain of HA2 (HFD) is considered to be conserved among different influenza subtypes [62]. HFD was described for their immunological potential and ability to induce antibodies [63]. Antibodies directed against HFD could inhibit the fusion between the viral and endosomal membrane and neutralize virus infectivity; however, no effective protection was reported exclusively due to HFD. It has been shown that HFD conjugated to keyhole limpet hemocyanin (KLH) has the ability to induce antibodies in mice following two immunizations and provide cross protection against homologous and heterologous influenza virus challenges [64]. On the other hand, HFD is small in size and was found to be less immunogenic than M2e [65]. Moreover, the alpha helix domain of HA2 (HαD), when tested as immunogen, induced antibodies with broader neutralizing activity and provided protection against influenza viruses of distinct subtypes [39, 43, 66]. The inclusion of HFD and HαD epitopes of the H5, H7, and H9 influenza viruses may further enhance the protection efficacy of ME-based vaccines.

In the present study, the immunization of mice with Ad-based ME vaccine failed to induce strong virus-neutralizing antibody responses. The Ad-H5ME vaccine failed to induce sterilizing immunity against high dose virus challenges, but our vaccine significantly reduced the virus replication in the lungs of mice challenged with antigenically distinct H5N2, H7N9, and H9N2 influenza viruses. The presence of heterosubtypic protection in the absence of strong neutralizing antibodies indicates the role of other players in providing cross-protection, including CMI responses and non-neutralizing humoral responses against M2e, HFD, and HαD. These findings are consistent with our previous results where the vaccination of mice with Ad-based vaccines expressing H1 HA induced high levels of broadly reactive, stalk-specific antibodies and reduced the lung viral titers of H5N1, H7N2, and H9N2 after challenges [50]. Similarly, Epstein et al. found that a DNA prime-Ad boost with the H1N1 NP induced CMI responses that protected mice against heterosubtypic challenges [67]. Two other studies of H3 HA Ad-based vaccine efficacy in mice and pigs have shown that protection against heterosubtypic challenges does not require the presence of neutralizing antibodies [29, 68].

It is known that HA518-specific CTL are induced in BALB/c mice following influenza virus infection, but these responses are less pronounced than NP147-specific CTL responses [69]. The role of HA518 epitope-specific CD8 T cells in heterologous or heterosubtypic protection is not clearly understood; however, our protection data from the mouse groups inoculated with Ad-H5HA, Ad-H7HA, or Ad-H9HA clearly suggest that HA518-specific CTL did not play a significant role in heterosubtypic protection.

NP is relatively conserved and represents a target for CTL-mediated immune responses critical to virus clearance and recovery after infection [40]. Hence, these CMI responses are cross-protective between different influenza A subtypes [70]. The TNP epitope included in our Ad-H5ME vaccine induced NP147 epitope-specific CD8 T cells secreting IFNγ in the vaccinated group. It is anticipated that NP147 epitope-specific CD8 T cells will contribute to the reduction of lung viral titers after virus challenges. This is consistent with the finding that the inclusion of TNP (NP147-155) with H5 HA in a DNA vaccine resulted in reducing the lung viral titers following a H5N1 challenge [71]. Additional studies have demonstrated that TNP was found to be immunogenic in mice, induced CD8 T-cell responses, and played a role in virus clearance [72, 73]. In our preliminary work we observed that mice immunized twice with 10^8^ PFU of an Ad vector expressing only the TNP resulted in approximately 0.4 log decreases in virus lung titers following challenge with an influenza virus compared to the animal group inoculated with the empty Ad vector [Ad-ΔE1E3]. These results suggest that in our ME vaccine study, humoral immune responses against M2e, HFD, and HαD domains contributed to approximately 1.1, 0.8, and 1.4 log reductions in lung virus titers against the H5, H7, and H9 influenza viruses, respectively. Additional studies will be required to evaluate the roles of individual epitopes in protection against the H5, H7, and H9 influenza viruses.

Pre-existing vector immunity is considered to impact vector uptake and to reduce the level and duration of transgene expression. We previously demonstrated that moderate levels of pre-existing vector immunity (<520 virus neutralizing titer) did not significantly hamper the protective efficacy of an Ad vector-based influenza vaccine in a mouse model [53]. Whereas, the higher levels of pre-existing vector immunity (1500 virus neutralizing titer) could be overcome by either varying the route of immunization or increasing the vaccine dose. Furthermore, several other approaches have been developed to circumvent the pre-existing vector immunity, including the use of other human Ad serotypes, non-human Ad, and DNA prime followed by an Ad boost. The impact of vector immunity on the immunogenicity and protective efficacy of Ad-H5ME vaccine should be measured in a separate study.

Future developments toward a universal influenza vaccine may include relatively conserved B cell domains comprised of HA2 and M2e, and conserved T cell domains of NP. Further studies will be required to evaluate the best strategy to allow the improved presentation and recognition of the epitopes on HA2 and M2e for the development of strong, cross-protective humoral immune responses. Additional experiments are necessary to determine an approach for the vaccine formulation containing NP, HA2, and M2e, which would result in balanced humoral and CMI responses as well as enhanced cross-protection against different influenza subtypes.
